# Cytomegalovirus Latency and Reactivation: An Intricate Interplay With the Host Immune Response

**DOI:** 10.3389/fcimb.2020.00130

**Published:** 2020-03-31

**Authors:** Eleonora Forte, Zheng Zhang, Edward B. Thorp, Mary Hummel

**Affiliations:** ^1^Department of Surgery, Comprehensive Transplant Center, Northwestern University Feinberg School of Medicine, Chicago, IL, United States; ^2^Department of Pathology and Pediatrics, Northwestern University Feinberg School of Medicine, Chicago, IL, United States

**Keywords:** cytomegalovirus, latency, reactivation, inflammation, oxidative stress, epigenetics

## Abstract

CMV is an ancient herpesvirus that has co-evolved with its host over millions of years. The 236 kbp genome encodes at least 165 genes, four non-coding RNAs and 14 miRNAs. Of the protein-coding genes, 43–44 are core replication genes common to all herpesviruses, while ~30 are unique to betaherpesviruses. Many CMV genes are involved in evading detection by the host immune response, and others have roles in cell tropism. CMV replicates systemically, and thus, has adapted to various biological niches within the host. Different biological niches may place competing demands on the virus, such that genes that are favorable in some contexts are unfavorable in others. The outcome of infection is dependent on the cell type. In fibroblasts, the virus replicates lytically to produce infectious virus. In other cell types, such as myeloid progenitor cells, there is an initial burst of lytic gene expression, which is subsequently silenced through epigenetic repression, leading to establishment of latency. Latently infected monocytes disseminate the virus to various organs. Latency is established through cell type specific mechanisms of transcriptional silencing. In contrast, reactivation is triggered through pathways activated by inflammation, infection, and injury that are common to many cell types, as well as differentiation of myeloid cells to dendritic cells. Thus, CMV has evolved a complex relationship with the host immune response, in which it exploits cell type specific mechanisms of gene regulation to establish latency and to disseminate infection systemically, and also uses the inflammatory response to infection as an early warning system which allows the virus to escape from situations in which its survival is threatened, either by cellular damage or infection of the host with another pathogen. Spontaneous reactivation induced by cellular aging/damage may explain why extensive expression of lytic genes has been observed in recent studies using highly sensitive transcriptome analyses of cells from latently infected individuals. Recent studies with animal models highlight the potential for harnessing the host immune response to blunt cellular injury induced by organ transplantation, and thus, prevent reactivation of CMV and its sequelae.

## Introduction

HCMV is a member of the betaherpesvirus family that infects 50–90% of the population, depending on age, socio-economic status, and country of origin (Cannon et al., 2010). Primary infection in immunocompetent individuals is typically sub-clinical, and is controlled by the host immune response, resulting in clearance of cells that are lytically infected and producing virus. However, in some cells, viral gene expression is shut off, and episomal viral genomes remain in the nucleus in a quiescent state called latency. Under appropriate conditions, this latent virus becomes activated and produces infectious virus. Reactivation of the virus is generally not associated with disease in immunocompetent individuals, but, under conditions of immunosuppression, such as those typically used to prevent rejection of transplanted organs or hematopoietic progenitor cells (HPCs), reactivation of latent virus is associated with increased risk for multiorgan disease, diminished graft survival, graft-vs.-host disease, infection with other pathogens, post-transplant lymphoproliferative disorders, and higher mortality (Griffiths and Lumley, 2014; Ljungman et al., 2014; Azevedo et al., 2015; Kaminski and Fishman, 2016; Fishman, 2017; Leeaphorn et al., 2019). The risk of CMV disease in transplant patients is dependent on the serostatus of the donor and recipient. In solid organ transplants, the donor positive, recipient negative combination poses the highest risk for complications. Without therapeutic intervention, CMV viremia occurs in up to 90% of these patients and symptomatic infection occurs in 50–60% of patients (Fishman, 2017). Improvements in diagnosis and anti-viral drugs targeting CMV replication have significantly improved transplant outcomes. In the era of modern anti-viral therapy, high risk patients are often given 3–6 months of prophylactic treatment with the viral kinase inhibitors valganciclovir or ganciclovir. However, late onset CMV disease may occur in 25–40% of patients after withdrawal of therapy (Fishman, 2017). Furthermore, all currently available drugs have significant drawbacks, including nephrotoxicity, neutropenia, and emergence of resistant strains, and CMV infection still has a negative impact on both patient and graft survival (Leeaphorn et al., 2019).

In addition, CMV infection can be transmitted from the mother to the fetus during pregnancy. The incidence of congenital CMV infection is estimated to be >30,000/year in the United States. 10–15% of these infants have clinically apparent disease at birth, with symptoms ranging from mild transient effects to severe multiorgan dysfunction, including jaundice, hepatosplenomegaly, microcephaly, chorioretinitis, and sensorineural hearing loss (SNHL). The prognosis for these severely affected infants is very poor. An additional 10–15% of infants who are infected, but do not have clinically apparent disease at birth, will develop late onset sequelae, primarily SNHL. Hearing impairment is the most common complication of congenital CMV infection, and accounts for 21–24% of cases hearing loss in young children (Manicklal et al., 2013). *In utero* transmission can occur both during primary infection and in mothers who have prior immunity, and experience new infection with another strain, or reactivation of latent virus. Because of the high prevalence of CMV infection in the population, nearly two thirds of congenitally infected infants are born to mothers with pre-existing immunity (James and Kimberlin, 2016). Thus, the widespread presence of latent CMV poses a hazard for the populations most at risk for CMV infection and its sequelae. The molecular mechanisms that allow the virus to establish latency and to reactivate are not well-understood, and further studies are needed to design interventions to protect these vulnerable populations.

### CMV Life Cycle

CMV infection is transmitted by contact with infectious body fluids, including blood, breast milk, saliva, urine, and genital secretions. Mucosal and glandular epithelial cells are permissive for infection, and these cells are the likely ports of entry and exit for the virus. Many other cell types, including connective tissue cells, smooth muscle cells, and vascular endothelial cells, as well as specialized cells within organs, including hepatocytes in the liver, alveolar epithelial cells in the lung and neuronal cells in the retina and brain, become lytically infected during the acute phase of infection (Sinzger et al., 2008). Thus, CMV establishes a systemic infection in the host, with many different cell types in all major organs affected. In contrast to most cell types, polymorphonuclear leukocytes and monocytes do not support lytic replication, but they do take up virus particles and express immediate early (IE) antigens (Grefte et al., 1994). These observations, combined with cell culture studies of HCMV-infected cells and studies with animal models, have led to the hypothesis that abortively infected PMNs or monocytes carry internalized virions through the blood to disseminate the virus to various organs (Gerna et al., 2000; Sinzger et al., 2008; Daley-Bauer et al., 2014; Stevenson et al., 2014). In the bone marrow, HCMV infects hematopoietic progenitor cells, where it establishes a lifelong latent infection. Although these cells are the progenitors for both lymphoid and myeloid cell lineages, latent virus is detectable only in monocytes in the blood. Thus, CMV occupies many different biological niches within the host at various stages of its life cycle, and the virus exploits immune cells of the myeloid lineage, both to achieve a disseminated infection during the acute phase of infection, and to establish a latent infection, where it can persist indefinitely.

### Experimental Models for Studying CMV Latency and Reactivation

HCMV infection of fibroblasts has been used for many years as experimental models to study lytic CMV replication, while models to study latency and reactivation have focused on infection of primary HPCs and monocytes. In addition, cell lines such as Kasumi-3 and THP-1 cells, which are more tractable models for myeloid progenitor cells and monocytes, respectively, and NTera2 cells, which have characteristics of committed neuronal progenitor cells, have been useful for studying latency and reactivation. However, studies of HCMV pathogenesis have been hampered by the fact that HCMV does not infect other species. For this reason, investigators have turned to primate or rodent models, using the related rhesus, rat and murine cytomegaloviruses. MCMV in particular has been useful in studying immune control of infection and mechanisms of reactivation. MCMV is similar to HCMV in mechanisms of immune evasion, epigenetic regulation of gene expression, and in ability to establish latency and to reactivate in response to organ transplantation.

### CMV Gene Expression and Function

Herpesviruses are ancient viruses. The three subfamilies, alpha, beta, and gamma herpesviruses, are thought to have arisen from a common ancestor as much as 400 million years ago, prior to the emergence of mammals 60–80 million years ago, and they have therefore coevolved with their hosts (Davison, 2011). All herpesviruses share the same tripartite temporal pattern of gene regulation, in which a small number of immediate early genes are initially expressed, followed by the early, and then the late genes (Mocarski, 2013). The IE-2 gene encodes a transcriptional transactivator protein that recruits cellular RNA polymerase II to early gene promoters to activate the second phase of transcription. Many of these genes encode enzymes required for replication of viral DNA, including the viral DNA polymerase. Expression of these genes permits amplification of viral DNA. Late viral transactivators (LVTs), which turn on expression of the late genes following DNA replication, are also expressed in the early phase of replication. Viral DNA replication and LVT expression permit the third phase of viral gene expression, leading to expression of the viral structural proteins and assembly of infectious viral particles. The 236 kbp CMV genome is the largest of the human herpesviruses, with the capacity to encode at least 165 genes, four non-coding RNAs and 14 miRNAs. Of the protein-coding genes, 43–44 are core replication genes common to all herpesviruses.

117 genes are dispensable for growth in fibroblasts, but they have roles in replication in other cell types and in immune evasion (Dunn et al., 2003; Yu et al., 2003). CMV encodes many immune evasion genes, which counteract the host immune response in two ways: (1) by interfering with the function of the innate and adaptive immune response, for example, by disabling presentation of cellular antigens to T cells and NK cells; (2) by interfering with immune effector functions, by expression of immunomodulatory cytokines such as viral IL10, disruption of cellular signaling pathways, or blocking apoptosis (Vossen et al., 2002; Smith and Khanna, 2013). The net result of this process in immunocompetent hosts is a balance between viral replication and host immune control that prevents uncontrolled viral spread and disease. However, CMV, like other herpesviruses, can also evade immune detection in some cells by silencing viral gene expression through establishment of latency.

### CMV Latency: Are There Latency-Specific Transcripts?

Latency is defined as a state in which replication-competent viral DNA is present, but infectious viral particles are not produced. However, there has been much confusion about the transcriptional status of latent viral genomes—whether they are transcriptionally quiescent, or express a small number of specific latency genes, or express many lytic genes, but do not produce virus, or produce virus below the level of detection of a plaque assay. Early studies seminal studies by Reeves and Sinclair using either naturally or experimentally infected monocytes or HPCs were consistent with repression of lytic transcripts in latency. These studies showed that the immediate early promoter region of the viral genome was heterochromatinized with hypoacetylated and H3K9-methylated histones and the repressive factor heterochromatin protein 1, and that IE transcripts were not detectable (Reeves et al., 2005a,b). Importantly, epigenetic reprogramming and reactivation of virus could be induced by differentiation of these cells to dendritic cells and stimulation with inflammatory mediators. Subsequent analyses have confirmed that CMV DNA is heterochromatinized in various experimental models of latency (Ioudinkova et al., 2006; Abraham and Kulesza, 2013; Lee et al., 2015; Rauwel et al., 2015; Gan et al., 2017).

Analyses of viral RNA expression identified a select group of genes that were expressed in latency in the absence of detectable IE gene expression, most notably, UL138 and LUNA, which is transcribed from the pp71 locus in an antisense direction (Bego et al., 2005; Goodrum et al., 2007; Reeves and Sinclair, 2010; Reeves and Compton, 2011; Lee et al., 2015). A number of other transcripts have been variably associated with latency, including US28, UL111A, a viral homolog of the immunosuppressive cytokine IL10, UL144, sense and antisense transcripts from the IE region, and non-coding RNAs 2.7 and 4.9 (Kondo et al., 1996; Beisser et al., 2001; Jenkins et al., 2004; Cheung et al., 2006; Poole et al., 2013). All of these genes are expressed during lytic infection of fibroblasts, as well as in latency models, and their expression is therefore not unique to the state of latency.

Genetic analyses have also indicated that some viral genes have a role in latency. Mutation of UL138 and US28 has no effect on viral gene expression or replication of the virus in permissive fibroblasts (Dunn et al., 2003; Yu et al., 2003; Goodrum et al., 2007; Umashankar et al., 2011; Humby and O'Connor, 2015). However, expression of lytic genes and replication of virus is higher in HPCs infected with UL138 or US28 mutants than wild-type viruses, indicating that these genes are negative regulators of viral replication in myeloid progenitor cells, which are a site of latency (Goodrum et al., 2007; Humby and O'Connor, 2015; Lee et al., 2015, 2016; Zhu et al., 2018). Although UL138 represses IE transcription and enhances methylation of H3K9 at the MIEP, it is not required for HDAC-independent repression of IE gene expression and is not sufficient to maintain latency in myeloid cells (Lee et al., 2015). Surprisingly, pUL138 also enhances the response to TNF–α, through up-regulation of cell surface expression of its receptor (Le et al., 2011). Since TNF-α can induce reactivation of CMV in some models (see below), these observations suggest a role for UL138 in both latency and reactivation. Mutation of miR-UL148D also disrupts latency, although different mechanisms have been reported (Lau et al., 2016; Pan et al., 2016).

Recent studies of viral gene expression have taken advantage of the development of sensitive, high throughput, and unbiased analyses to interrogate the latent viral transcriptome in both naturally and experimentally infected myeloid cells (Rossetto et al., 2013; Cheng et al., 2017; Shnayder et al., 2018; Schwartz and Stern-Ginossar, 2019). In contrast to earlier reports, these studies detected expression of many lytic genes, and did not find evidence for a latency-specific transcriptional profile. The observation that expression of many lytic genes is detectable in latently infected PBMCs of healthy, seropositive donors is difficult to reconcile with epigenetic studies showing heterochromatinization of the viral genome in experimental models of latency (Rauwel et al., 2015).

Single cell analysis of experimentally infected monocytes has shed some light on this paradox (Shnayder et al., 2018, 2020; Schwartz and Stern-Ginossar, 2019). This study revealed that there is marked heterogeneity in the cellular response to infection. In many cells, expression of many lytic genes was initially detectable, although virus production was not detected. The number of viral genes expressed in each cell became progressively lower over time, consistent with transcriptional repression. However, there was considerable intercellular variability in the kinetics of repression of viral gene expression, and a small minority of cells retained expression of late lytic genes throughout the course of infection. These observations are consistent with the hypothesis that there is a transient activation of viral gene expression, followed by repression, in most cells. Our own studies with the Kasumi-3 latency model showed that lytic genes are initially expressed, but are then repressed over time, and genes that have been associated with latency, including UL138, US28, and RNA2.7 are repressed in parallel with lytic genes during establishment of latency (Forte et al., 2018). Detection of many viral transcripts in healthy seropositive donors may therefore reflect reactivation of latent virus and transcription of viral genes in newly infected cells. Further analyses of the viral epigenome, both in bulk populations and at the single cell level, are needed to better understand the state of the viral transcriptome in latency.

### Cellular Tropism and Viral Gene Expression

The repertoire of viral genes expressed is dependent on the cell type. Previous studies have demonstrated organ-specific expression of rat CMV RNAs *in vivo*, and cell type-specific expression of HCMV RNAs *in vitro*, suggesting that the cellular environment markedly influences the viral transcriptional program (Streblow et al., 2007; Towler et al., 2012; Van Damme and Van Loock, 2014). The cell type also influences the functional effects of genes on viral replication. Many of the genes that are dispensable for growth in fibroblasts have roles in other cell types. For example, HCMV encodes four genes with homology to human chemokine receptors, US27, US28, UL33, and UL78, with cell type specific roles in infection (Krishna et al., 2018). pUL78 is dispensable for growth in fibroblasts, but is required for entry into epithelial cells and for efficient viral replication in endothelial and epithelial cells (O'Connor and Shenk, 2012; Krishna et al., 2018). pUS28 which is a negative regulator of viral replication in myeloid cells, facilitates cell-to-cell spread of the virus in epithelial cells (Noriega et al., 2014). The UL133–138 region encodes several genes with complex roles in regulation of viral replication in different cell types (Umashankar et al., 2011; Bughio et al., 2013, 2015; Caviness et al., 2016; Goodrum, 2016). In addition to genes that negatively regulate replication in myeloid cells, CMV encodes several genes that suppress viral replication in other cell types, e.g., UL9, UL20a, UL23, and UL30 in fibroblasts; UL10 and UL16 in epithelial cells; US16 and US19 in HMVECs (Dunn et al., 2003). These observations suggest that different biological niches may place competing demands on the virus, such that genes that are unfavorable in some contexts are favorable in others. Despite their negative effects on replication in some cells, these genes may confer an adaptive advantage to the virus by enhancing systemic infection.

The interplay between the cellular environment and the virus is most striking in cells of the hematopoietic system. CD34+ HPCs in the bone marrow harbor latent viral DNA *in vivo* (Mendelson et al., 1996; Sindre et al., 1996; Zhuravskaya et al., 1997; Hahn et al., 1998), and CMV is able to enter and infect HPCs in cell culture models. However, there are differences between subpopulations of progenitor cells: CD34^+^/CD38^−^ cells support an HCMV infection with the hallmarks of latency, but a subset of CD34^+^/CD38^−^ cells expressing a stem cell phenotype (lineage^−^/Thy-1^+^) support productive HCMV infection (Goodrum et al., 2004). The receptors for entry of the virus may differ among cell types, and this may affect the outcome of infection (reviewed in Collins-McMillen et al., 2018). While platelet-derived growth factor receptor (PDGFR) is important for entry into fibroblasts, endothelial cells, and epithelial cells, epidermal growth factor receptor (EGFR) and integrins are important receptors for entry of the virus into HPCs and monocytes (Chan et al., 2009; Kabanova et al., 2016; Kim et al., 2017). Signaling through these receptors is thought to prime the cells for latency through activation of phosphatidylinositol-4,5-bisphosphate 3-kinase (PI3K) signaling, down-regulation of IE1/2 expression, up-regulation of UL138, and modulation of cytokine expression (Buehler et al., 2016; Peppenelli et al., 2016; Kim et al., 2017; Collins-McMillen et al., 2018).

The MIEP is less active in HPCs than fibroblasts, and the viral transactivator pp71 is unable to translocate into the nucleus to activate the MIEP (Saffert et al., 2010). In addition to the MIEP, IE transcripts can be initiated at one of two intronic promoters, iP1 and iP2. These promoters are active in both fibroblasts and myeloid cells, but there are cell type-specific differences in their relative utilization (Arend et al., 2016; Collins-McMillen et al., 2019). Following infection, there is an initial burst of lytic gene expression, which is subsequently repressed to allow establishment of latency, both in primary HPCs and in Kasumi-3 cells (Goodrum et al., 2002, 2004; Cheung et al., 2006; O'Connor and Murphy, 2012; Rauwel et al., 2015; Forte et al., 2018; Zhu et al., 2018; Galinato et al., 2019).

HPCs are the progenitors for both lymphoid and myeloid cells in peripheral blood. Although latent viral DNA has not been detected in mature lymphoid cells, CMV DNA is detectable at very low frequency in monocytes isolated from healthy seropositive donors (Taylor-Wiedeman et al., 1991, 1994; Bolovan-Fritts et al., 1999; Slobedman and Mocarski, 1999; Parry et al., 2016; Jackson et al., 2017). Early studies indicated that viral genes were not expressed in infected monocytes (Stevenson et al., 2014), but more recent studies show that viral gene expression in experimentally infected monocytes follows the same pattern of activation of lytic gene expression followed by repression observed in HPCs (Hargett and Shenk, 2010; Rossetto et al., 2013; Shnayder et al., 2018, 2020). Thus, demonstration that the experimental criteria for latency have been met (repression of viral gene expression and lack of virus production) is an important consideration when analyzing CMV latency. An increase in the relative abundance of early genes, such as UL138 and US28, over immediate early genes is often used as a criterion for establishment of latency. However, this analysis should be viewed with caution, since these changes also occur during lytic infection.

Expression of viral genes substantially alters the phenotype of both infected monocytes and HPCs. Expression of US28 in infected HPCs reprograms the cells into immunosuppressive monocytes (Zhu et al., 2018). The reported effects of HCMV infection on monocytes include increased lifespan through induction of anti-apoptotic genes (Chan et al., 2010; Reeves et al., 2012; Collins-McMillen et al., 2015; Peppenelli et al., 2016), re-programming the cells to an M1 or M2-like macrophage phenotype (Smith et al., 2004; Chan et al., 2008, 2012; Avdic et al., 2013; Stevenson et al., 2014), modulation of cell signaling pathways (Kew et al., 2017; Krishna et al., 2017) evasion of killing by neutrophils (Elder et al., 2019), and induction of an anergic-like state (Shnayder et al., 2020).

Differentiation of monocytes to dendritic cells prior to infection increases the permissiveness of the cells (Riegler et al., 2000; Hertel et al., 2003; Huang et al., 2012; Hertel, 2014; Sinclair and Reeves, 2014; Coronel et al., 2015). Collectively, the data on experimentally infected hematopoietic cells, which shows 1) that viral gene expression is first turned on and then turned off in monocytes and HPCs, and 2) that differentiation of these cells increases their permissiveness, suggest that undifferentiated myeloid cells and monocytes express repressor(s) that shut off transcription of viral genes in a cell type- and differentiation-specific manner.

### What Shuts Off CMV Gene Expression in Myeloid Cells?

The mechanisms by which CMV gene expression is repressed to establish latency in myeloid cells are largely unknown. It is possible that repression is simply a host defense mechanism, and the virus does not play an active role in this process. There is considerable evidence that host intrinsic immunity recognizes histone-free viral DNA penetrating the nuclear pore complex and ND10 proteins act to chromatinize viral genomes and inactivate gene expression at the outset of infection (reviewed in Kalejta, 2013). However, these responses occur in permissive cells, and are not specific to myeloid cells. Furthermore, recent studies show that, although ND10 proteins restrict lytic HCMV replication in fibroblasts and reactivation in hematopoietic cells, they do not serve as key determinants in establishment of latency (Wagenknecht et al., 2015; Poole et al., 2018).

**Figure 1:**
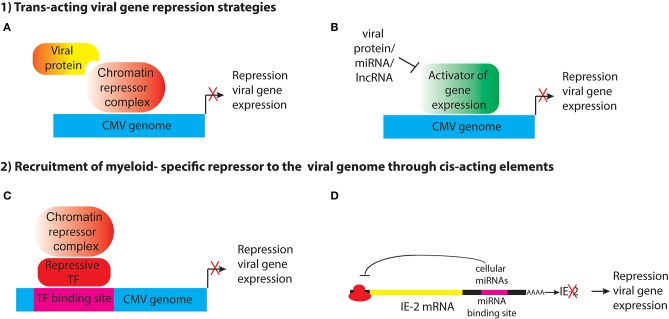
Potential strategies for establishment of HCMV latency in myeloid progenitor cells through the action of (1) trans-acting viral factors or (2) cis-acting elements encoded by the viral genome that recruit myeloid-specific repressors to viral genes. **(A)** Viral proteins such as pUL138 and pUS28 may act indirectly to alter histone modifications at the MIEP to maintain repression of viral genes. **(B)** pUS28 attenuates activation of c-fos and NF-κb, which activate the MIEP. HCMV also encodes miRNAs that target viral and cellular genes. Viral lncRNAs could also act to block activation of viral gene expression. **(C)** The viral genome may contain binding sites for myeloid-specific repressive transcription factors that recruit co-repressor complexes to silence viral gene expression. **(D)** Viral RNAs have binding sites for cellular miRNAs that inhibit viral gene expression.

Ability to establish latency through transcriptional repression likely provides a survival advantage to the virus, by allowing it to evade immune detection and persist in the host in the face of a highly effective adaptive immune response, and so the virus may have evolved mechanisms to shut off its gene expression in specific cell types. This could be achieved through different strategies, including (1) expression of *trans-acting* viral genes that represses viral gene expression through recruitment of cellular enzymes that heterochromatinize the genome, either directly or indirectly; (2) encoding *cis-acting* elements that recruit cellular repressors to the viral genome (Figure 1). These strategies will be discussed below.

Expression of viral protein(s) that modulate IE gene expression (Figure 1A). An example of this is pUL138, which has been shown to enhance methylation of H3K9 at the MIEP and repress IE transcription in THP1 cells (Lee et al., 2015). Because it is localized to the Golgi apparatus, rather than the nucleus, pUL138 must act indirectly to modify histones bound to the MIEP. In addition, trans-acting factors could interfere with signaling pathways that lead to activation of the MIEP (Figure 1B). pUS28, a cell surface G-protein coupled receptor, attenuates activation of c-fos and NF-κB, which activate the MIEP, and prevents reprogramming of the viral chromatin in monocytic THP1 cells (Krishna et al., 2017, 2019). Expression of a protein to establish latency poses a number of challenges for the virus. First, the protein would need to avoid recognition by the host adaptive immune response. Second, unless it is an immediate early gene, it would need to be expressed in the absence of the IE proteins that are normally required to activate subsequent phases of viral gene expression. Third, the protein would have to be expressed only in cells where latency is established, or it would have to have cell type-specific functions. pUL138 and pUS28 have apparently acquired some or all of these functions (Tey et al., 2010; Mason et al., 2013; Krishna et al., 2017, 2018).

A variation of this approach, which circumvents immune detection issues, would be to encode microRNAs or other non-coding RNAs to achieve transcriptional repression (Figure 1B). HCMV encodes 14 miRNAs, which target cellular genes involved in cell cycle control, immune evasion, apoptosis, and the secretory pathway that controls virion assembly during lytic infection (Hook et al., 2014). Several HCMV-encoded miRNAs have been reported to be expressed in latency, including miR-UL112-1, which targets the 3′UTR of UL123 (IE1), as well as cellular defense genes and miR-UL148D, which targets the chemokine RANTES and inhibits activin A-triggered secretion of IL-6 (Grey et al., 2007; Murphy et al., 2008; Nachmani et al., 2009; Kim et al., 2012; Lee et al., 2012; Fu et al., 2014; Shen et al., 2014; Lau et al., 2016; Meshesha et al., 2016; Pan et al., 2016). However, microRNAs function by preventing translation or enhancing degradation of their target mRNAs, and they are not thought to have a direct role in heterochromatinization of viral genomes in latency.

It has long been postulated that the host cell environment plays an important role in establishment of latency (Reeves and Sinclair, 2008; Sinclair and Reeves, 2014). Thus, an alternative strategy for the virus to establish latency would be to encode *cis*-acting DNA elements that recruit cellular repressors to the viral genome (Figure 1C). Since transcription factor binding sites require relatively short DNA sequences, which could be tucked into non-coding regions, this would be a very efficient way for the virus to acquire the ability to establish latency. If the repressor was expressed only in specific cell types, such as myeloid progenitors and monocytes, the virus could establish latency specifically in these cells without interfering with lytic replication in other cell types that do not express the repressor(s).

Although cellular repressors that negatively regulate the MIEP, such as YY1 and ERF, have been previously identified (Liu et al., 1994; Bain et al., 2003; Wright et al., 2005) myeloid-specific repressor(s) required for transcriptional silencing of the HCMV genome have yet to be discovered. However, a possible clue has come through the study on the role of KAP1 (KRAB-associated protein 1, also known as TRIM28 and TIF1b) in CMV latency (Rauwel et al., 2015). KAP1 is a transcriptional co-repressor of the N-terminal tripartite motif (TRIM) family that represses expression of endogenous retroviruses and transposable elements. Studies from the Trono lab demonstrated that KAP1 was required for transcriptional repression, recruitment of the H3K9 methyltransferase SetDB1, and deposition of the repressive H3K9me3 histone mark onto the viral genome in HPCs (Rauwel et al., 2015). KAP1 is a multifunctional protein with separate domains that mediate nuclear localization, interaction with transcription factors, oligomerization, and regulation of transcription (Iyengar and Farnham, 2011), and it is subject to a variety of post-translational modifications that determine its binding partners and function (Cheng et al., 2014). KAP1 SUMOylation recruits SETDB1 and the nucleosome and remodeling deacetylase (NuRD) complex to silence gene expression (Ivanov et al., 2007; Zeng et al., 2008; Iyengar and Farnham, 2011). KAP1 can also be phosphorylated on Ser824, and this results in loss of binding to SETDB1 and repressor function (Li et al., 2007). Studies from the Trono lab showed that phosphorylation of KAP1 acts as switch to turn on CMV gene expression and reactivate latent virus in HPCs (Rauwel et al., 2015).

KAP1 does not bind to DNA directly, but rather, is recruited onto DNA by interaction between the TRIM domain of KAP1 and the Kruppel-associated box (KRAB) domain of KRAB-ZNF proteins. Expression of KAP1 is not limited to myeloid progenitor cells. Thus, KAP1 itself is not a myeloid-specific repressor, but these observations raise the intriguing possibility that a myeloid-specific KRAB-ZNF protein could mediate binding of KAP1 to *cis*-acting elements in the CMV genome to repress viral gene expression and establish latency in HPCs and monocytes. 28 KAP1 binding sites were identified in the HCMV genome, but no consensus site that would suggest the identity of the repressor could be determined from these sequences. Surprisingly, none of the KAP1 binding sites were in the MIEP. Epigenetic regulation of MIEP activity is considered to be a major factor controlling lytic infection, latency, and reactivation. Although KAP1 is known to mediate long-range transcriptional repression though heterochromatin spreading (Groner et al., 2010), these observations raise the possibility that additional myeloid-specific repressors could be involved in establishment of CMV latency. Further studies are needed to confirm the role of KAP1 in CMV latency, to identify the proteins that mediate recruitment of KAP1 onto the viral genome, and to identify additional factors that could directly mediate transcriptional silencing of CMV DNA in myeloid lineage cells.

In addition to KAP1, previous studies identified Gfi-1 as a repressor of the HCMV MIEP and as a negative regulator of HCMV lytic replication in fibroblasts (Zweidler-Mckay et al., 1996; Sourvinos et al., 2014). Gfi-1 is a Zn-finger transcriptional repressor that acts through recruitment of the CoREST/LSD1 histone-modifying complex (Saleque et al., 2007). Although Gfi-1 is expressed in some non-hematopoietic cells, it has an important role in cell fate decisions in the hematopoietic system by promoting lymphoid and neutrophil differentiation at the expense of myeloid cells (van der Meer et al., 2010; Moroy et al., 2015). Thus, Gfi-1-binding elements in the MIEP may have an important role of repression of HCMV gene expression in myeloid progenitor cells.

In a variation on the theme of *cis*-acting elements that mediate HCMV latency, the virus could encode targets for cellular microRNAs that repress viral gene expression (Figure 1D). The 3′UTR of UL122 (IE2) encodes a target sequence for the cellular miRNA hsa-miR-s200, which mediates repression of protein expression. Mutant viruses lacking this sequence fail to establish latency (O'Connor et al., 2014). IE2 encodes a transcriptional transactivator that recruits Pol II to early gene promoters to activate early gene expression. Parida et al. proposed that IE2 expression is required to prevent deposition of nucleosomes and chromatin-mediated gene silencing (Parida et al., 2019). Additional studies have identified other cellular microRNAs that maintain latency (Poole et al., 2011). However, it is unclear whether these miRNAs are expressed specifically in myeloid cells.

### Are There Non-hematopoietic Sites of Latency?

While it is well-accepted that hematopoietic progenitor cells in the bone marrow are one of the sites of CMV latency, the cell type harboring latent virus in solid organs is more controversial. CMV infection is transmitted at a high frequency following transplantation of a donor-positive organ into a seronegative recipient. Donor HPCs in the bone marrow are clearly not the source of transmission in this scenario. Because the frequency of CMV-positive cells in the blood is very low, and often below the detection level for even the most sensitive assays (Parry et al., 2016; Jackson et al., 2017), peripheral blood mononuclear cells are also unlikely to be the source of transmission. Tissue resident macrophages or dendritic cells in the donor are potential sites of latency (Sinclair and Reeves, 2014), although this has not been demonstrated. Endothelial cells are known to be sites of latency in organs of MCMV latently infected mice (Koffron et al., 1998; Seckert et al., 2009). HCMV encodes two genes, US16 and US19, that are negative regulators of replication in microvascular endothelial cells, and may therefore have a role in establishment of latency in these cells (Dunn et al., 2003). A previous study did not identify latently infected endothelial cells in the saphenous veins of seropositive subjects (Reeves et al., 2004). However, endothelial cells are highly heterogeneous with respect to function and gene expression (Aird, 2007a,b; Marcu et al., 2018). Sinusoidal endothelial cells and peritubular capillary endothelial cells were identified as sites of MCMV latency in the liver and kidney, respectively (Koffron et al., 1998; Seckert et al., 2009). These sites have not been investigated, and the sites of HCMV latency in human organs therefore remains an open question. This issue is important because it bears on the mechanisms of latency and reactivation: are there endothelial cell type specific factors controlling latency and reactivation in solid organs? Further studies of this question are needed.

### Reactivation of Naturally Acquired HCMV Infection

In clinical settings, reactivation of HCMV has long been associated with sepsis and other systemic inflammatory conditions in patients who are not immunosuppressed (Docke et al., 1994; Cook et al., 1998; Kutza et al., 1998; Heininger et al., 2001; Limaye et al., 2008; Kalil and Florescu, 2009; Walton et al., 2014), with allograft rejection in immunosuppressed recipients of solid organ transplants (Grattan et al., 1989; Reinke et al., 1994; Lao et al., 1997; Lautenschlager et al., 1997; Evans et al., 2001; Razonable et al., 2001; Nett et al., 2004; Dmitrienko et al., 2009), and with graft vs. host disease in stem cell transplant recipients (Lonnqvist et al., 1984; Meyers et al., 1986; Bostrom et al., 1990; Matthes-Martin et al., 1998; Broers et al., 2000; Boeckh and Nichols, 2004). Treatment with antilymphocyte antibodies, which is often used to control rejection of solid organs, is a known risk factor for reactivation of CMV (Hibberd et al., 1992; Fietze et al., 1994; Portela et al., 1995). All of these conditions are associated with high systemic levels of inflammatory cytokines, particularly TNF-α, as well as diminished immune function. TNF-α activates the MIEP and the transcription factor NF-κB, which has multiple binding sites in the MIEP (Stein et al., 1993; Docke et al., 1994; Fietze et al., 1994; Prosch et al., 1995). These observations led several investigators to propose the hypothesis that graft rejection, infection with other pathogens, or antilymphocyte treatment could lead to reactivation of CMV through NF-κB-mediated activation of the MIEP. This would set up an amplifying loop, in which viral infection increases expression of MHC genes, leading to increased immunogenicity of the graft and higher expression of inflammatory cytokines, which further drives reactivation and rejection (Fietze et al., 1994; Fishman and Rubin, 1998; Hayry et al., 1998; Reinke et al., 1999).

Although clinical disease is typically apparent only in patients who are immunosuppressed or suffering acute illness, there is considerable evidence that reactivation occurs frequently in healthy seropositive adults, and that it may exacerbate chronic illnesses. Shedding of CMV DNA was detected in one or more body sites, including the nose, skin, oral cavity, and vagina, in 7–8% of asymptomatic adults (de Franca et al., 2012; Wylie et al., 2014). Furthermore, analyses of the CMV-specific T cell response in healthy adults and in mice latently infected with murine cytomegalovirus (MCMV) are consistent with repeated stimulation of the adaptive immune response due to reactivation of the virus (O'Hara et al., 2012; Klenerman and Oxenius, 2016). Interestingly, the frequency of detection of CMV DNA or antigenemia is significantly higher in patients with atopic dermatitis and periodontitis (Docke et al., 2003; Chalabi et al., 2008). It is well-established that CMV accelerates atherosclerosis, both in cardiac transplant patients and in animal models of transplantation, and CMV infection is a suspected etiologic agent in cardiovascular disease and mortality in the population at large (Streblow et al., 2008; Simanek et al., 2011; Kaminski and Fishman, 2016; Wang et al., 2017; Lebedeva et al., 2018).

The frequency of CMV reactivation increases with age (Stowe et al., 2007; Furui et al., 2013; Parry et al., 2016). Although this has often been attributed to immune senescence associated with aging, an increased inflammatory response due to impaired health or age-related inflammation (“inflammaging”) (Franceschi et al., 2018) may also contribute to reactivation of HCMV in the elderly (Vescovini et al., 2010; Jackson et al., 2017).

### *In vivo* Models for Studying CMV Latency and Reactivation

Rodents latently infected with the related viruses, rat, and murine cytomegalovirus (RCMV and MCMV), have been useful in studying CMV pathogenesis and immune control (Reddehase et al., 2008; Streblow et al., 2008). These models offer several advantages over cell culture models, including the ability to use different strains of inbred mice as donor/recipient pairs and to use genetically deficient mice to define specific pathways that contribute to reactivation. Unlike cell culture systems, these models permit the study of latency and reactivation in the presence of an intact immune response. Most important, they can be used to study reactivation of latent CMV in experimental conditions that mimic clinical settings, including organ transplantation.

### Epigenetic Silencing of MCMV Gene Expression in Latency

Analyses of MCMV gene expression in latency showed that, although transcripts from the IE region were sometimes detectable, most genomes were transcriptionally silent in organs of latently infected mice (Kurz et al., 1997, 1999; Koffron et al., 1998; Kurz and Reddehase, 1999; Hummel et al., 2001). However, CpG dinucleotides in MIEP region of MCMV DNA are not methylated in latently infected mice, suggesting that the genome is not permanently inactivated (Hummel et al., 2007). As with HCMV, the latent MCMV genome is heterochromatinized with densely packed nucleosomes marked by de-acetylated and H3K9-methylated histones, and repressors, including HDACs, YY1, HP1-1γ, CBF1/Rbjk, CIR, and Daxx, are recruited onto the genome (Liu et al., 2008).

### Organ Transplantation Induces Both Antigen-Specific and Non-specific Injury

Organ transplantation initiates a complex cascade of events leading to injury and, in the absence of immunosuppression, to rejection of the donor organ. During the process of organ transplantation, the blood supply to the donor organ is interrupted, and then restored when the organ is grafted into the recipient. This causes a non-specific injury known as ischemia/reperfusion injury (I/R) (reviewed in Eltzschig and Eckle, 2011; Braza et al., 2016). Hypoxia during the ischemic phase causes damage to mitochondrial electron transport chains and production of reactive oxygen and nitrogen species (ROS and RNS). The resulting loss of ATP production leads to increased anaerobic metabolism and dysfunction of ion pumps, which causes cellular swelling and reduction in intracellular pH, which in turn affects enzymatic activity. Loss of antioxidants and mitochondrial injury results in further oxidative damage following restoration of the blood supply in the reperfusion phase, which leads to endothelial cell dysfunction, DNA damage, and release of molecules with damage-associated molecular patterns (DAMPs) that activate innate immunity and promote infiltration of neutrophils. These cells release ROS and other effector molecules that further damage the organ. Generation of intracellular ROS due to I/R injury also leads to activation of redox-sensitive transcription factors, including NF-κB and AP-1, which regulate expression of cellular genes that mediate protection against oxidative stress, cell surface adhesion molecules that mediate attachment and diapedesis of inflammatory cells and inflammatory cytokines that further activate the cells (Karin and Shaulian, 2001; Gloire et al., 2006; Morgan and Liu, 2011; Taniguchi and Karin, 2018).

Unless organ transplants are performed on identical twins, human transplant donors and recipients have a mismatch in the major histocompatibility genes, which are recognized as foreign antigens by recipient immune cells. This activates an adaptive immune response, leading to infiltration of T cells that mediate rejection of the graft. In animal models, the respective roles of the adaptive immune response and I/R injury can be distinguished by using different combinations of inbred strains of mice as donors and recipients. Both the adaptive immune response and I/R injury are induced when the donor and recipient have an MHC mismatch (allogeneic transplant), but only I/R injury is present when the recipient is genetically identical to the donor (syngeneic transplants).

### Allogeneic Transplantation Activates the MIEP Through Epigenetic Reprogramming

Early studies showed that transplantation of latently infected kidneys into naïve allogeneic, immunocompetent recipients induced transcriptional reactivation of IE gene expression within 2 days post-transplant (POD2) (Hummel et al., 2001). In contrast, reactivation was not observed when latent kidneys were transplanted into syngeneic recipients. Transcriptional reactivation correlated with expression of inflammatory cytokines, including TNF-α, IL-1, and interferon gamma, and activation of transcription factors with binding motifs in the MIEP, including NF-κB and AP-1. In parallel studies, transplantation of kidneys from transgenic mice carrying a *lacZ* reporter gene under the control of the HCMV MIEP (MIEP-*lacZ* mice), induced activation of the reporter. In addition, activation of the MIEP could be induced by treatment of MIEP-*lacZ* mice with TNF-α alone (Hummel et al., 2001). The rapid transcriptional response observed in latently infected mice suggested that reactivation of IE gene expression was stimulated by the process of organ transplantation, rather than simply unmasking pre-existing expression through the loss of immune surveillance. This was demonstrated unequivocally in MIEP-lacZ mice, where the reporter was an endogenous gene. Analyses of changes in the viral epigenome induced by allogeneic transplantation further strengthened this hypothesis by showing that transplantation induced recruitment of RNA polymerase II and transcription factors NF-κB and AP-1 onto the MIEP, and replacement of repressive histone marks with activating modifications (Liu X. F. et al., 2010, 2013,2016).

Further studies with MIEP-lacZ and MCMV latently infected mice were performed in an effort to unravel the pathways that drive reactivation of CMV gene expression at early times post-transplant. Although TNF-α was expressed in allogeneic transplants and TNF-α was sufficient to induce reactivation of IE gene expression in the lungs of latently infected mice, studies with TNF receptor (TNFR)-deficient MIEP-*lacZ* mice or MCMV latent TNFR-deficient mice showed that renal transplant-induced activation of the MIEP could occur independently of TNF-α signaling (Simon et al., 2005; Zhang et al., 2008, 2009). In addition, I/R was sufficient to induce activation of the MIEP in MIEP-*lacZ* transgenic mice, and this was also independent of TNFR signaling (Kim et al., 2005). Transcription factor analyses revealed that NF-κB and AP-1 were activated in response to I/R independently of TNFR signaling. Thus, these studies showed that multiple inflammatory insults, including TNF-α and oxidative stress, can contribute to transcriptional reactivation of IE gene expression.

### Ischemia/Reperfusion Injury Is Sufficient to Induce Reactivation of MCMV

Although reactivation of IE gene expression was observed at POD2 in allogeneic transplants, later stages of viral replication were not observed, either at POD2 or later times, up to POD8. However, complete reactivation of infectious virus, with systemic spread to other organs of the recipient, was observed 4–8 weeks after transplantation of latently infected kidneys into immunodeficient NOD-*scid*IL2Rg^null^ (NSG) mice (Li et al., 2012). These observations highlight the role of immune surveillance in limiting reactivation of the virus *in vivo*. Because NSG mice are deficient in B and T cells, as well as NK cells, these studies demonstrated that an adaptive immune response to foreign antigens was not required for reactivation of latent MCMV. Recent studies have provided further insight into mechanisms of reactivation. These studies showed that reactivation and systemic spread of the virus can be induced when immunocompetent allogeneic recipients are pharmacologically immunosuppressed, using a combination of anti-lymphocyte serum to deplete T cells, the calcineurin inhibitor FK506 to block T cell activation, and the steroid dexamethasone to inhibit activation of NF-κB (Zhang et al., 2019). This protocol is very similar to regimens currently used in clinical settings. Reactivation was detectable as early as POD7, with increasing viral load at POD14 and POD28. In contrast to previous studies analyzing reactivation of IE gene expression at POD2, reactivation was also observed when syngeneic mice were used as the recipients, and the signal was amplified by immunosuppressing the recipient and harvesting the organ at POD28. Importantly, no reactivation was observed when latently infected mice were treated for 28 days with immunosuppression alone. Flow cytometric, proteomic and transcriptome analyses demonstrated that the immunosuppression protocol was effective in blocking the adaptive immune response, including infiltration of T cells and activation of pathways associated with host innate and adaptive immune responses, including Th1/Th2 cells, allograft rejection, co-stimulation and B cell development, Toll-like receptor signaling and cytokine signaling. However, pathways associated with oxidative stress and DNA damage were up-regulated in the donor kidney, regardless of whether the recipients were immunosuppressed. These studies therefore demonstrated that oxidative stress associated with I/R injury was sufficient to induce reactivation of MCMV, and that the inflammatory signaling pathways activated by the adaptive immune response were not required.

### Sepsis Induces Reactivation of MCMV

Reactivation of HCMV is strongly associated with sepsis (Kutza et al., 1998; Walton et al., 2014), a systemic inflammatory response to bacterial infection characterized by both proinflammatory responses initiated by recognition of PAMPs, and immunosuppression (Angus and van der Poll, 2013). PAMPs signal through Toll-like receptors, NODs, NLRs, and RIGs to activate transcription factors that regulate expression of interferons and inflammatory cytokines, including NF-κB, AP-1, and IRFs (Ishii et al., 2008). Both the MCMV and HCMV MIEPs have binding sites for NF-κB and AP-1, and thus mimic the promoters of many genes involved in the cellular innate immune response (Hummel and Abecassis, 2002; Kropp et al., 2014). Studies with MCMV have shown that cecal ligation and puncture (CLP), a procedure that models sepsis, induces a systemic response that results in reactivation of MCMV in the lungs of latently infected mice (Cook et al., 2002).

Collectively, the studies with latent MCMV and MIEP-lacZ transgenic mice are consistent with the idea that reactivation in response to organ transplantation is initiated by a transcriptional stimulus that flips a switch and reprograms the viral chromatin from an “off” to an “on” state. This switch can be triggered by inflammatory cytokines whose expression is induced by the allogeneic response to foreign antigens in the organ, such as TNF-α, by the systemic inflammatory response induced by bacterial infection, or by injury sustained during ischemia and reperfusion of transplanted organs. These insults activate signaling pathways that lead to activation of the MIEP and reprogramming of the viral epigenome. Immunosuppression of the recipient leads to full blown reactivation of infectious virus, and systemic infection of the recipient. The signaling pathways that lead to activation of the MIEP in this context are active in many different cell types, and they are not specific to myeloid cells.

### Reactivation of HCMV in Hematopoietic Cells

Reactivation of HCMV can be induced by various stimuli in different models of experimental latency in hematopoietic cells. A common theme of many of these stimuli is activation of inflammatory signaling or DNA damage response pathways. In primary CD34+ HPCs or CD14+ monocytes, reactivation can be induced by differentiation to dendritic cells and stimulating those cells with inflammatory mediators, including IL-6 and LPS (Reeves et al., 2005a,b; Reeves and Compton, 2011; Huang et al., 2012). LPS, a bacterial cell wall component, is a PAMP that binds to the pathogen recognition receptor TLR4, activates NF-κB and AP-1, and induces expression of IL-6, TNF-α, and IL1β (Ishii et al., 2008; Tanaka et al., 2014). These cytokines have pleiotropic effects that orchestrate the acute phase response in the liver, as well as innate and adaptive immune responses to infection (Tanaka et al., 2014; Kalliolias and Ivashkiv, 2016; Mantovani et al., 2019). LPS-induced reactivation of HCMV in monocyte-derived dendritic cells occurs through IL-6, which mediates activation of the ERK/MSK/CREB pathway and reprograms histones bound to the MIEP (Kew et al., 2014; Dupont et al., 2019).

In addition, recent studies show that chloroquine, which activates a DNA damage response, can induce reactivation of HCMV in primary HPCs, and this effect is potentiated by co-treatment with TNF-α (Rauwel et al., 2015). Chloroquine activates ATM, which is the master regulator of the response to double-stranded DNA damage. This results in ATM-dependent phosphorylation of KAP1 bound to the HCMV genome (Rauwel et al., 2015). As noted above, phosphorylation of KAP1 causes a switch in binding partners, resulting in relaxation of the chromatin to facilitate repair, and de-repression of gene expression (Ziv et al., 2006; Li et al., 2007; Iyengar and Farnham, 2011). In the Kasumi-3 model, reactivation can be induced by treatment with inflammatory cytokines TNF-α and IL-1, or by treatment with the chemical TPA (O'Connor and Murphy, 2012; Forte et al., 2018). Treatment of Kasumi-3 cells with TNF-α induces both a DNA damage response, including phosphorylation of H2AX, ATM, and KAP1, as well as activation of NF-κB (Forte et al., 2018).

In monocytic THP1 cells, reactivation can be induced by treatment with TPA, which induces the cells to differentiate into macrophage-like cells (Beisser et al., 2001; Arcangeletti et al., 2016). TPA binds to and activates PKC (Castagna et al., 1982). It has pleiotropic effects, including activation of AP-1, oxidative stress, and DNA damage, as shown by activation of ATM and phosphorylation of H2AX, activation of MSK, and phosphorylation of histone H3 (Tanaka et al., 2006; Teng et al., 2009). The mechanisms by which TPA induces reactivation of HCMV, and the link between reactivation and differentiation in these cells have not been investigated. As with other stimuli, TPA likely induces reactivation in part through modification of histones bound to viral DNA (Gan et al., 2017).

### The MIEP Is a Complex Region Activated by Inflammatory Signals

**Figure 2:**
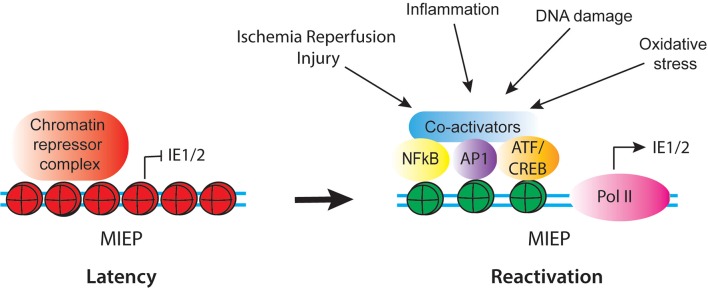
Schematic of regulatory factors that control activity of the CMV MIEP in latency and reactivation. In latency, the MIEP is occupied by histones with repressive modification (red) and chromatin repressor complexes. Reactivation is triggered by reprogramming of the MIEP, such that repressors are replaced by activating transcription factors and co-activators and histones bound to the MIEP acquire activating modification (green). Multiple stimuli associated with inflammation and cellular injury can lead to activation of the MIEP.

The MIEP is thought to be the master regulator of latency and reactivation. In latently infected cells, the MIEP is heterochromatinized, and myeloid-specific factors likely play a role in recruiting repressors onto the viral genome to achieve cell type specific latency. Reactivation of latent HCMV likely requires replacement of these repressors with activating transcription factors, remodeling of viral chromatin to increase accessibility of the MIEP to transcription factors, and reprogramming of histones bound to the MIEP (Figure 2). The MIEP has binding sites for multiple transcription factor families, including Elk-1, SRF, Sp1, NF-κB, CREB, and AP-1. Analysis of HCMV mutants has demonstrated that some of these elements enhance replication under some conditions, but are redundant in others (Keller et al., 2003, 2007). For example, the NF-κB sites are dispensable for viral replication in actively growing fibroblasts, but are essential in quiescent cells (Gustems et al., 2006; Caposio et al., 2007). In addition, some transcription factors act co-operatively with others under specific circumstances. For example, NF-κB acts cooperatively with CREB and AP-1 during lytic infection of fibroblasts, and synergizes with CREB to activate the enhancer during reactivation of latently infected N-Tera2 cells (Lashmit et al., 2009; Caposio et al., 2010; Liu X. et al., 2010; Isern et al., 2011; Yuan et al., 2015). Recent studies show that intronic promoters primarily drive reactivation of IE gene expression in myeloid cells (Collins-McMillen et al., 2019). These promoters are activated by TPA in THP1 cells and by co-culture with fibroblasts in media containing a cocktail of inflammatory cytokines (IL-6, G-CSF, and GM-CSF) in primary hematopoietic progenitor cells (Collins-McMillen et al., 2019).

Several of the transcription factor families that bind to the MIEP are composed of multiple combinations of factors, which are activated by a diverse array of stimuli, including growth factors, inflammatory mediators, DNA damage and oxidative stress, which act through multiple, independent signaling pathways. Thus, the combination of stimuli and downstream factors that could potentially activate the MIEP is extraordinarily complex. This likely allows the virus to (re)activate gene expression in a wide variety of circumstances (Figure 2). In some contexts, such as IL-6 mediated activation of the MIEP, differentiation of myeloid cells is required to render the cells responsive to the stimulus (Reeves and Compton, 2011; Huang et al., 2012; Dupont et al., 2019). However, differentiation is not required for reactivation under other circumstances, such as DNA damage or exposure to TNF-α (Rauwel et al., 2015; Forte et al., 2018). Both IL-6 and TNF-α are expressed in response to injury and infection. Thus, while HCMV latency is established through exploitation of the myeloid-specific transcription program, reactivation occurs in response to a broader array of signals. These signals are activated by inflammation, DNA damage, and cellular injury.

### Can We Target and Educate the Host Immune Response to Prevent Reactivation of CMV?

A long-standing goal of the transplant community is to wean off the dependence of chronic broad-spectrum T cell immunosuppressants and to establish donor antigen-specific tolerance to the graft. This approach would in theory preserve the efficacy of the adaptive immune response to pathogens, including antiviral immunity, while also preventing rejection of the organ due to alloimmunity. This is an important consideration, especially in the face of the potential for CMV antiviral drug-resistance. Humoral immunity is particularly critical in the setting of graft-vs.-host disease, in that CMV reactivation can be prevented after transplant by transfer of immune serum (Martins et al., 2019). Multiple translational tolerance strategies are under current investigation and vary in their approach, route of administration, and molecular or cellular target. Discussion of this vast topic is beyond the scope of this review, however, the following citations are provided for the interested reader (Luo et al., 2016; Zuber and Sykes, 2017; Gupta et al., 2019). Transplant immune tolerance has been achieved in pre-clinical small and large animal models, and in certain human patients. An obstacle to transplant tolerance is that CMV itself can impair the mobilization of cells that are required for transplantation tolerance (Dangi et al., 2018). In addition, age is a risk factor in that patients older than 65 years may be of heightened risk for CMV reactivation (Hemmersbach-Miller et al., 2019). Future studies are therefore necessary to examine comprehensive clinical efficacy with respect to control of CMV in these settings. Separately, strategies that target perioperative tissue injury and innate cellular and cytokine inflammation due to allograft ischemia and reperfusion after organ implantation, as discussed above, may also yield new therapeutic approaches that work together with immune tolerance through combinatorial therapies. Continued research into the basic science and mechanisms of CMV reactivation under the aforementioned conditions is warranted to identify new molecular therapeutic targets.

## Conclusions

During their long period of co-evolution, CMV and its host have engaged in an arms race, in which the virus exploits vulnerabilities in the host, which then adapts by developing new ways to detect infection, block viral replication, and kill infected cells, and viruses counteract these defenses by developing new strategies to evade detection and killing. CMV has evolved to exploit the hematopoietic system in various ways. It uses myeloid cells to achieve a disseminated infection in the host. In addition, CMV also exploits the transcriptional program that regulates hematopoietic cell differentiation to repress viral gene expression specifically in myeloid progenitor cells. This allows the virus to escape immune detection and establish a latent infection in cells with a long lifespan, without compromising its ability to replicate in other cell types. There may be additional sites of latency, such as endothelial cells, and it is tempting to speculate that similar mechanisms may control establishment of latency in these cells or their progenitors.

By definition, production of infectious virus does not occur in latency. Survival of the virus is therefore dependent on survival of the host, and the ability to escape from a host or a cell whose survival is compromised would be expected to confer a significant adaptive advantage. Data from both clinical studies, as well as experimental studies using MCMV-infected mice and HCMV-infected hematopoietic cells suggests that CMV reactivates in response to cellular injury and the systemic inflammatory response that occurs during infection with other pathogens. These signaling pathways are active in many different cell types, and are not restricted to myeloid cells. Targeting these pathways may lead to new therapies to prevent reactivation of CMV and its sequelae.

## Author Contributions

MH wrote the manuscript. ET, EF, and ZZ contributed ideas and edited the manuscript. EF made the figures.

### Conflict of Interest

The authors declare that the research was conducted in the absence of any commercial or financial relationships that could be construed as a potential conflict of interest.
